# Addressing Microbial Resistance Worldwide: Challenges over Controlling Life-Threatening Fungal Infections

**DOI:** 10.3390/pathogens12020293

**Published:** 2023-02-10

**Authors:** Leonardo Martins-Santana, Caroline Patini Rezende, Antonio Rossi, Nilce Maria Martinez-Rossi, Fausto Almeida

**Affiliations:** 1 Department of Genetics, Ribeirão Preto Medical School, University of São Paulo, Ribeirão Preto 14040-900, Brazil; 2 Department of Biochemistry and Immunology, Ribeirão Preto Medical School, University of São Paulo, Ribeirão Preto 14040-900, Brazil

**Keywords:** fungal infections, candidiasis, cryptococcosis, aspergillosis, SARS-CoV-2

## Abstract

Fungal infections are a serious global concern because of their ability to spread and colonize host tissues in immunocompromised individuals. Such infections have been frequently reported worldwide and are currently gaining clinical research relevance owing to their resistant character, representing a bottleneck in treating affected people. Resistant fungi are an emergent public health threat. The upsurge of such pathogens has led to new research toward unraveling the destructive potential evoked by these species. Some fungi—grouped into *Candida*, *Aspergillus*, and *Cryptococcus*—are causative agents of severe and systemic infections. They are associated with high mortality rates and have recently been described as sources of coinfection in COVID-hospitalized patients. Despite the efforts to elucidate the challenges of colonization, dissemination, and infection severity, the immunopathogenesis of fungal diseases remains a pivotal characteristic in fungal burden elimination. The struggle between the host immune system and the physiological strategies of the fungi to maintain cellular viability is complex. In this brief review, we highlight the relevance of drug resistance phenotypes in fungi of clinical significance, taking into consideration their physiopathology and how the scientific community could orchestrate their efforts to avoid fungal infection dissemination and deaths.

## 1. Resistance—A Life-Threatening Human Condition

Several pathogenic and opportunistic fungal species can cause fungal disease. It affects over a billion people on Earth, particularly immunocompromised individuals, and is responsible for the death of millions of people worldwide [1,2]. A critical issue regarding the severity of such diseases is the ability of fungi to present or acquire resistance against drug therapies used to fight these infections.

Drug resistance is an evolving phenotype that confers the fungi ability to maintain their viability in the presence of chemical agents that cause damage. This may be due to a series of physiological mechanisms exhibited by the fungi to prevent cell death [3]. To address this, it is pivotal for clinical therapies to impose strategies capable of preventing fungal survival in host environments, as their lack could potentially result in fungal dissemination and host tissue colonization.

Several fungal species are considered of human clinical relevance because of their potential to cause diseases. Due to the broad spectrum of fungi capable of causing diseases, infection niches and symptoms may vary. Among such clinical conditions, it is noteworthy to mention Histoplasmosis caused by *Histoplama capsulatum* [4], Coccidioidomycosis [5], infections caused by Fusarium fungi [6], conditions related to Rhizopus infections [7,8], Emergomycosis [9], Candidiasis caused by Candida species (such as *C. albicans*, *C. auris*, *C. glabrata*, *C. krusei*, *C. tropicalis*, and others) [10], Aspergillosis (caused by different species of Aspergillus gender, such as *A. fumigatus*, *A. flavus*, *A. terreus*, and others) [11,12,13], Cryptococcosis [14], and dermatophytosis caused by different species [15].

Some fungal isolates present resistant traits, not being affected by antifungal drugs. These are being studied to discover new approaches to accomplish treatment success [16,17,18,19,20,21]. This is concerning since drug therapies have shown to be less effective against some clinical isolates, raising the critical call for interventions that enable efficient treatment of fungal diseases [22,23]. Antifungal drugs are distributed among distinct classes, such as azoles (fluconazole, itraconazole, ketoconazole, etc.), echinocandins (caspofungin, anidulafungin, micafungin), polyenes (amphotericin B, nystatin, etc.), and allylamines (terbinafine) applied in clinical therapies to eliminate the fungal burden in infected individuals [24,25].

Although it is hard to avoid and eliminate the fungal burden from niches, decontamination measures, such as chemoprophylaxis, air filtration with HEPA filters, cleaning hospital surfaces, and several others could be applied to prevent fungal diseases [26,27,28].

**Figure 1:**
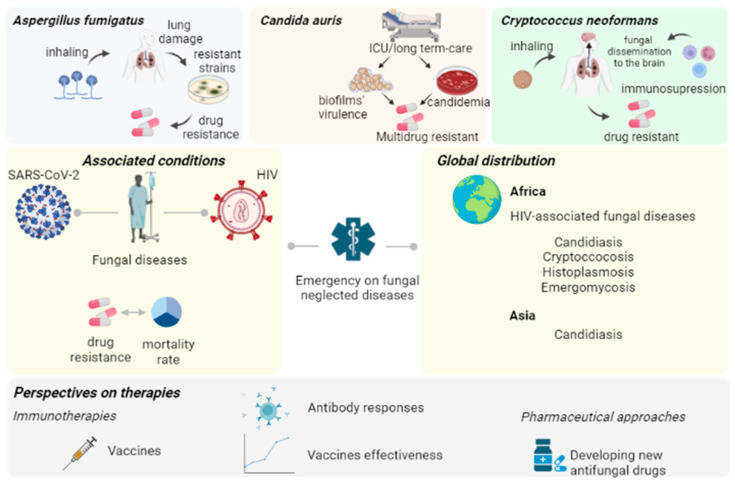
Schematic representation of the contagious and development of fungal diseases in humans. Aspergillosis, candidiasis, and cryptococcosis threaten health systems worldwide due to their resistant character. Previous conditions may be associated with these diseases, which makes them harder to treat. Human Immunodeficiency Virus (HIV), cancer, and long-term ICU patients are more likely to develop critical clinical conditions. SARS-CoV-2-associated infections are being reported worldwide. Therefore, perspectives comprising vaccines and searching for new drug therapies to treat fungal diseases are expected. Created with BioRender.com.

This brief review summarizes the critical points regarding pathogenic fungi of clinical relevance concerning scientists worldwide. Here, we discuss recently published data covering aspects of infections caused by three pathogens that directly impact human health owing to the resistant phenotypes they have acquired. *Candida auris*, *Cryptococcus neoformans*, and *Aspergillus fumigatus* are the central points of our commentaries since the importance of these species in clinical conditions are enormous (Figure 1). Table 1 shows that infections caused by these three pathogens are distributed across Earth. They are directly related to underlying conditions—such as Human Immunodeficiency Virus (HIV), cancer, and long-term care in hospitals—affecting thousands of people worldwide. These species are often neglected, particularly in Africa.

### 1.1. Candida auris

*Candida* genus harbors several species reported that cause candidiasis and systemic infections characterized as candidemia. *C. auris* is an emergent pathogen that has frequently been a target of extensive research due to its multidrug resistance [29,30]. It was first discovered in Japan in 2009 [31]. Over the years, infections caused by this yeast have been reported worldwide [29]. Such diseases are related to nosocomial circumstances being reported highly in intensive care unit (ICU) patients, patients who were under the use of venous catheters, antibiotic therapies, and surgical procedures, and individuals who present with previous health conditions, such as diabetes and immunocompromised individuals [32,33]. *C. auris* likely shows a preference for skin surface colonization at the expense of colonizing other biological niches [34]. Despite its predominant feature of colonization, bloodstream infections (candidemia) caused by this pathogen have already been reported [35], highlighting the threat of fungal infections. However, studies have detected this yeast in the axilla, nares, and groin [36]. Mortality rates caused by this pathogen are high, up to 70%, particularly in cases of candidemia [37,38].

A crucial aspect of *C. auris* infection is its ability to form biofilms, sessile cell communities embedded in a polymeric extracellular matrix composed primarily of polysaccharides, which can easily adhere to surfaces [39]. This type of cellular organization makes cells less susceptible to antifungal action, increasing the resistance of this pathogen when cells aggregate into thin layers [40]. Biofilms constitute a perfect example of virulence factors in *C. auris* that directly influence the destructive potential of this fungus to cause damage since this pathogen can form biofilms with a higher virulence capacity [41].

Evidence of whether the pathogenic potential of *C. auris* is due to the presence of virulence factors is described in a study by Larkin et al. (2017) [42]. By researching 16 isolates of *C. auris*, the authors showed that 37.5% and 64% of the analyzed strains produced phospholipase and proteinase, respectively, in a strain-dependent manner. They also observed a reduced ability of the isolates to respond to fluconazole and amphotericin B [42].

In a meta-analysis and systematic review, Sekiere (2018) reported that 44.29%, 15.46%, 12.67%, and 3.48% of isolates were resistant to fluconazole, amphotericin B, voriconazole, and caspofungin, respectively [43]. The reduced susceptibility to antifungals is one of the most concerning phenotypes and has led to the search for new drug therapies for *C. auris*. One of the cellular mechanisms that support such phenotypes is the occurrence of mutations in target genes related to resistance. For example, mutations in genes related to ergosterol biosynthesis, such as *erg11*, result in amino acid substitutions in target proteins that can drive resistance in isolates of this pathogen [44,45]. Furthermore, mutations in genes that participate in the synthesis of glucans, such as *fks1*, have been identified in *C. auris* echinocandin-resistant strains [46]. Notably, transcriptomics also appears to be involved in stress responses in the presence of caspofungin and amphotericin B, as Gao et al. (2021) detected the upregulation of a long non-coding RNA known as DINOR in *C. auris* under these conditions.

**Table 1:** Cases of Candidemia, Invasive Aspergillosis, and Cryptococcosis in people worldwide. NUC—Non underlying condition or others. RD—Respiratory Disorder. ISP—Immunossupression (related to transplantation/chemotherapy). ICU—Intensive Care Unit (critical care). Rate/100 K—Rate of cases per 100,000 people.

	Candidemia						Invasive Aspergillosis						Cryptococcosis						Reference
	NUC	HIV/AIDS	Cancer/ISP	RD	ICU/surgery	Rate/100 K	NUC	HIV/AIDS	Cancer/ISP	RD	ICU/surgery	Rate/100 K	NUC	HIV/AIDS	Cancer/ISP	RD	ICU/surgery	Rate/100 K	
**Africa**																			
Algeria			1414		606	5			47	2818		7.1		28	7			0.09	[47]
Cameroon			779		334	5			134		1041	5.3		6720				30	[48]
Egypt			2889		1238	5			664		8337	10.7		38				0	[49]
Kenya			1393		597	5			239			0.6		11,900				29	[50]
Malawi			600		150	5		1080	106			6.7		8200				47.3	[51]
Mozambique			925		396	5			159			0.6		18,640				70.5	[52]
Namibia			87		37	5		108	15	1	259	15.4		543				21.8	[53]
	**Candidemia**						**Invasive Aspergillosis**						**Cryptococcosis**						
	NUC/other	HIV/AIDS	Cancer + ISP	RD	ICU + surgery	Rate/100 K	NUC/other	HIV/AIDS	Cancer + ISP	RD	ICU + surgery	Rate/100 K	NUC/other	HIV/AIDS	Cancer + ISP	RD	ICU + surgery	Rate/100 K	
**America**																			
Argentina			1096		1097	5			334	268	1934	5.8		372	60			1	[54]
Brazil	11,654 *	870 *	3131 *		13,336 *	249 *			6813		1851	4.47	138	6694				3.52	[55]
Dominican Republic														29				0.29	[56]
Peru			1090		467	5			438	1183		5		156				0.5	[57]
	**Candidemia**						**Invasive Aspergillosis**						**Cryptococcosis**						
	NUC	HIV/AIDS	Cancer/ISP	RD	ICU/surgery	Rate/100 K	NUC	HIV/AIDS	Cancer/ISP	RD	ICU/surgery	Rate/100 K	NUC	HIV/AIDS	Cancer/ISP	RD	ICU/surgery	Rate/100 K	
**Asia**																			
Bangladesh			5670		2430	5			972		4194	3.2		15				0.01	[58]
China			65,609		16,402	5.72		1040	31,800	1,145,908		82.21							[59]
India			150,427		37,607	13.5		2358	7040	1885	239,651	18	1844	9682				0.83	[60]
Indonesia			20,030		6680	10		1400	2700	900	44,500	18.6	340	7540	790			8.7	[61]
Korea	1522				455	4.12							38	6				0.09	[62]
Kyrgyzstan					250	4.2			46		246	4.9		25				0.4	[63]
Malasya			1073		460	5			184		834	3.3	47	700	108			2.8	[64]
Pakistan									777		10,172	5.9							[65]
Tajikistan					371	4.2			27		256	3.2		41				0.5	[66]
	**Candidemia**						**Invasive Aspergillosis**						**Cryptococcosis**						
	NUC	HIV/AIDS	Cancer/ISP	RD	ICU/surgery	Rate/100 K	NUC	HIV/AIDS	Cancer/ISP	RD	ICU/surgery	Rate/100 K	NUC	HIV/AIDS	Cancer/ISP	RD	ICU/surgery	Rate/100 K	
**Europe**																			
Azerbaijan					499	5			81	36	577	7		5				0.05	[67]
Belgium			388		165	5			402		273	6.08							[68]
Greece			379		162	5			85		1040	10.4		2				0.02	[69]
Serbia			50		468	7.3								5				0.07	[70]
Sweden		1	30		97	4.7			98	108	89	3							[71]

* Candidemia in hospitalized patients in Brazil.

The authors concluded that DINOR is a virulence factor and involved in stress responses, which broadens the perspectives for future studies on long non-coding RNAs toward drug resistance in this pathogen [72].

*C. auris* remains an enigmatic microorganism for the scientific community since it is essential to correlate its pathogenic potential with host immunology-triggered responses. Little is known about how this pathogen can evade the immune system and evoke host defenses, leading scientists worldwide to research whether the mortality caused by it is related the most to its great pathogenic potential or its weak immune response.

### 1.2. Cryptococcus neoformans

*C. neoformans* is the foremost pathogen causing cryptococcosis. It can be found in several environmental niches—such as soil, trees, animals, and pigeon droppings [73]. Inhalation is the primary route of infection that spreads from the lungs to the central nervous system, particularly when cellular immunity is compromised [74]. Epidemiological data have allowed us to conclude that infection is often associated with an asymptomatic latent state [75,76]. Since the first clinical case description, cryptococcosis related to immunosuppression has been observed. Infection or reactivation is often fatal in immunosuppressed individuals with pneumonia and meningoencephalitis [77,78]. Cryptococcal diseases present antifungal resistance owing to the unique characteristics of this fungus, such as genomic plasticity and physiological adaptability [79]. Some authors have reported the use of therapies involving three classes of antifungals: polyenes, flucytosine, and azoles, which vary according to the location and severity of the disease, as well as the immune status of the host [78]. Echinocandins are a class of antifungals that inhibit the synthesis of β-glucan in the fungal cell wall by non-competitive inhibition of the enzyme β-1, 3-glucan synthase [80]. However, resistance to echinocandins in *C. neoformans* is almost not noticeable because of the low concentration of β-glucan in the cell wall of this fungus, which could make this drug ineffective against *C. neoformans* [81]. Genetic screening showed that echinocandin resistance is mediated by calcineurin signaling (Crm1 pathway), which is over-stimulated by *cdc50* deletion, resulting in cell death [82]. Other common resistance mechanisms involve alterations in *erg11* expression and the *afr1* efflux pump, an ABC transporter responsible for pumping fluconazole out of cells [83,84]. Studies have shown that fluconazole mediated-damaged could occur with high doses of this drug when *erg11* is overexpressed, as overexpression may increase the production of the target-enzyme concentration [85] In contrast, the overexpression of *afr1* increased resistance. This increase in *afr1* promotes the pumping of fluconazole out of the cell with less drug effect [83]. The ability of *C. neoformans* to undergo morphological transitions is exemplified by the formation of titan cells, which exhibit changes that contribute to antifungal resistance [86]. Titan cells have a thick cell wall and a highly reticulated capsule [87]. They can also generate daughter cells that are more adapted to the host environment and are more resistant to fluconazole [86]. Gerstein et al. (2015) showed that daughter cells derived from *C. neoformans* titan are more resistant to oxidative stress, similar to those used by the host immune system [88]. They produce daughter cells resistant to this antifungal agent in the presence of fluconazole, which suggests that morphological variations of titan cells may be a mechanism for generating genomic plasticity that leads to resistance to antifungal drugs [88]. A recent study by Carlson et al. (2021) showed that environmental conditions, such as nutrient deprivation and high temperatures, could influence antifungal sensitivity [89]. The authors observed that nutrient deprivation of fungal cells decreased susceptibility to fluconazole, whereas increasing the temperature improved treatment efficacy with fluconazole and amphotericin B [89]. Therefore, antifungal resistance mechanisms involve a complex interaction between environmental conditions, virulence factors, and changes in gene expression.

### 1.3. Aspergillus fumigatus

*A. fumigatus* is a ubiquitous filamentous fungus that efficiently disseminates due to its ability to form conidia. This life form can be readily inhaled by humans, resulting in the development of pulmonary diseases. Aspergillosis is a broad spectrum of infections caused by *Aspergillus* and may be clinically presented as the occupancy of bronchopulmonary cavities by spores (conidia) of this pathogen. This occupancy affects more dramatically neutropenic and immunocompromised individuals [90]. Furthermore, it can assume distinct clinical manifestations depending on the interaction between the host and pathogen, which groups these presentations into specific clinical condition sets [90]. Invasive pulmonary aspergillosis (IA), one of the most studied invasive manifestations of the disease, frequently presents as fever, hemoptysis, cough, shortness of breath, and chest pain [91]. On the contrary, there may be no symptoms in neutropenic or immunocompromised individuals because of the deficiency of the inflammatory response [91].

It is worth mentioning that despite the occurrences of aspergillosis in immunocompetent individuals being considerably lower compared with immunocompromised individuals, it has been possible to observe a higher prevalence of IA in non-neutropenic patients who are simultaneously facing underlying conditions, especially those in ICUs [92,93]. In addition, recent studies have attempted to shed light on the occurrence of IA in patients with respiratory illnesses, such as those affected by SARS-CoV2 [94,95]. Unfortunately, due to the high mortality rates of IA patients, scientific efforts must be made to circumvent such catastrophic scenarios [96]. Drug resistance may be a major enemy in the fight against IA. *A. fumigatus*, causative of IA, is of particular relevance to the *Aspergillus* species due to its occurrence. In some countries, using fungicides in agriculture can give rise to resistant strains, as *A. fumigatus* could become resistant to azoles by fungicide use [97,98]. This represents a massive threat to human health because their spores could be inhaled by immunocompromised individuals and thus pose a risk to this group of people. In this sense, the use of agricultural fungicides might contribute to increased resistance in *Aspergillus* [99,100]. Therefore, combatting *Aspergillus* drug-resistant strains is a scary but unavoidable duty of researchers worldwide.

A series of cellular mechanisms can confer azole resistance to *A. fumigatus*. Mutations in the gene *cyp51*, coding for an enzyme of the ergosterol biosynthesis pathway, are among the most studied mechanisms for generating resistant phenotypes [98]. Other mechanisms involved in ergosterol biosynthesis—such as mutations in the gene *hmg1*, coding for the enzyme HMG-CoA reductase—have been recently reported to be associated with triazole resistance in this pathogen [101,102]. As azole is the first-line drug treatment against antifungal diseases, resistance-associated phenotypes of isolates could be detrimental to the success of clinical therapies. Therefore, echinocandins could be used to circumvent such bottlenecks. As new strains resistant to antifungals are increasing, IA must be considered a clinical condition worthy of careful assistance since it could be fatal in some patients. A mutation in the gene *fks1* of *A. fumigatus* was recently described as a result of previous in vitro exposure of this fungus to an echinocandin drug, anidulafungin [103]. Satish and Perlin (2019) proposed a model of echinocandin resistance in which no mutations in the *fks1* gene were observed. In their study, in vitro exposure of *A. fumigatus* to caspofungin elicited mitochondrial reactive oxygen species production, resulting in alterations of the membrane lipid microenvironment of glucan synthase, which would lead to a decrease in the affinity between the enzyme and drug, contributing to the rise of a resistance phenotype in this fungus [104].

## 2. Exacerbated Fungal Infections in Association with the COVID-19 Pandemic

The pandemic caused by the new coronavirus SARS-CoV-2, responsible for the COVID disease, brings to discuss challenges that face our understanding of how potential secondary infections could be hazardous to public health worldwide. Long-term patients in the ICU and those receiving treatment in hospitals are more likely to be infected with pathogens. However, this scenario has been affected by the COVID-19 pandemic in recent years.

Infections caused by the yeast *C. auris* have been observed worldwide and have become a severe global threat due to the resistance traits exhibited by this pathogen. Countries such as Brazil, the United States of America, India, Lebanon, and Turkey have identified *C. auris* co-infections in COVID-19 patients [105,106,107,108,109]. Incidences of such infections in hospitalized patients could be critical for their health, especially in the presence of *C. auris*, which causes candidemia. Bloodstream infections would be the worst scenario for ill COVID patients, as the severity of infection could likely result in long-term care in hospitals or even the death of sick individuals. Furthermore, it could worsen the clinical conditions of such patients in cases where resistant strains of *C. auris* would be the source of secondary infections. In this sense, Chowdhary et al. (2020) observed that all *C. auris* isolates studied in their research were resistant to fluconazole, and 40% of them were resistant to amphotericin B [106], which highlights the concern of resistant strains in co-infections.

Aspergillosis is another secondary disease often diagnosed in ill COVID patients. A retrospective case series study by Martins et al. (2021) showed that three COVID patients were co-infected with aspergillosis, four presented candidemia, and one was co-infected with cryptococcosis [110]. The potential for fungi to co-infect individuals in nosocomial niches is clear. In such cases, the treatment of both clinical conditions must be rationally designed to guarantee survival. In this context, it could be challenging to treat both fungal infection and COVID because we know that using corticosteroids could constitute a risk factor for aspergillosis [111,112]. It is noteworthy that the deadly potential of *Aspergillus* coinfections is in hospitalized COVID patients. In addition, it is reasonable to discuss how drug therapies could be crucial to preserving human life.

Resistant fungi are likely to represent a threat to drug therapies in the case of COVID co-infections. Mohamed et al. (2021) described a fatal COVID-19 pneumonia case complicated by a multi-triazole-resistant *A. fumigatus* co-infection [113]. Genotyping analysis of the isolate identified a mutation in *cyp51A*, confirming the resistant phenotype of the pathogen, supported by the high Minimum Inhibitory Concentration (MIC) values for voriconazole, itraconazole, and posaconazole [113]. Resistant co-infections represent a considerable challenge for treating COVID patients since such infections are more likely to arise in nosocomial niches, where the severity of the clinical conditions of the ill patients is notable.

It is worth mentioning that other co-infections between fungi and SARS-CoV-2 have also been reported, such as the occurrences of mucormycosis, caused by species of Mucor gender, in several countries. These co-infections present high mortality rates and demand attention from global health [114,115,116].

In a world affected by the COVID-19 pandemic, scientific efforts should preserve life and reduce risks, especially those of drug therapies, with the utmost efficiency. In the meantime, with the expected end of the pandemic, the discussion to be braved relies on research for new drug discoveries and awareness to avoid neglecting fungal diseases.

## 3. Immunotherapeutic Approaches

Antifungal immunity is exerted by innate and adaptive immune cells and depends on factors such as the host’s immune status, fungal morphology and virulence, cell wall complexity, and site of infection [117]. Although innate immunity initiates the antifungal response, the interaction between the host and fungus is essential for initiating the adaptive immune response [118]. Cellular immunity is mediated by T lymphocytes that directly or indirectly control fungal proliferation and are classified as helper CD4 T cells and cytotoxic CD8 T cells [119]. Activated T cells secrete a set of cytokines that promote the differentiation of naïve T cells into subsets of T helper (Th) cells: Th1, Th2, Th17, and regulatory T cells (Tregs) [119].

Studies have been conducted to improve the antifungal arsenal by identifying new drug targets. However, the development rate of new antifungals offered by the market is lower than the increase in drug resistance [120]. Thus, alternative approaches involving the use of combination therapy, the development of new treatments, and the modulation of the host immune response are being explored [120]. A promising approach is the development of preventive immunotherapies and vaccines against fungal pathogens [121]. Conjugated vaccines based on fungal cell wall β-glucans have proven to be a broad-spectrum strategy to restrict infection and prolong survival in murine infection models by *Candida* spp., *Cryptococcus* spp., and *Aspergillus* spp. [122,123,124]. A new vaccine against *C. auris* was designed using an in silico approach with the selection of the adhesion protein agglutinin 3 (*als3*) involved in virulence and directed to predict T and B cell epitopes [125]. The results of the study showed that vaccine construction was stable, soluble, antigenic, and non-allergic, and exhibited stable interaction with the Toll-like receptor (TLR) and the major histocompatibility complex (MHC). In addition, it can be cloned and expressed in *Escherichia coli* [125]. Thus, it is possible to infer that the vaccine could be used as an alternative therapy for treating *C. auris*. However, its efficacy and safety still need to be determined through in vivo studies [125].

Vaccine development for infections caused by *C. neoformans* was first described by Devi et al. (1991), in which the use of the antiphagocytic antigen present in the fungal capsule—known as glucuronoxylomannan (GXM)—was able to induce an immune response without the help of T cells [126]. However, although vaccinated mice developed a specific anti-*Cryptococcus* antibody response, most did not show a protective immune response [126]. Current studies have identified 11 cryptococcal protein antigens (*cpd1*, *glo1*, *blp4*, *sacch*, *mep1*, *3143*, *4874*, *cda1*, *cda2*, *cda3*, and *sod1*), which conferred protection in BALB/c and C57BL/6 mice against challenge with *C. neoformans* strain KN99 when formulated in vaccines based on the encapsulation of glucan particles [127,128]. Further studies are needed to determine whether other adjuvants can replace glucan particles and the immune mechanism. In infections caused by *A. fumigatus*, vaccine therapy induced protective immunity in immunosuppressed and immunocompetent patients [129]. In a study by Rayens et al. (2021) in a murine model of drug-induced immunosuppression, a recombinant protein vaccine of *A. fumigatus* (AF.KEX1) generated a robust immune response and promoted a decrease in mortality and fungal load in the lungs of vaccinated mice [130]. Other studies have shown that conidia, mycelium extracts, or fungal culture filtrates can also induce adequate protection against aspergillosis [131,132].

Immune checkpoint therapy is an emerging immunotherapeutic strategy that targets the axis programmed cell death protein 1 (PD-1) and its ligand PD-L1, as this protein is a key regulator of the immune system. Its interaction with PD-L1 promotes suppression of the immune system [117]. A fungal infection study with *A. fumigatus* showed that PD-L1, mediated by α-(1,3) glycans, is a negative regulator of the immune response promoting the increased polarization of regulatory T cells [133]. Therefore, PD-L1 blockade efficiently inhibited the polarization of regulatory T cells mediated by α-(1,3) glycans and promoted a protective Th1 immune response in the host [133]. In addition, PD-1 blockade improved survival and reduced the fungal burden in a murine model of pulmonary aspergillosis [134]. Thus, exploring therapeutic approaches involving the PD-1/PD-L1 axis may be promising for improving protective immunity in human models.

In summary, we highlight some of the most encouraging therapeutic approaches for developing new treatments to combat current and emerging fungal threats. Furthermore, in the future, the greatest challenge regarding studies on fungal diseases will be to test the use of immunotherapy in clinical trials.

## 4. Perspectives

As previously discussed, one of the bottlenecks in the treatment of fungal diseases is the high prevalence of resistant strains worldwide. It is essential to understand that the availability of antifungal drugs may not be the same for all countries worldwide since economic measures may require interchangeable measures according to government priorities.

Fungal diseases are not democratically distributed. The higher rates of candidemia are related to occurrences in middle-income countries since 50% of the global cases of candidemia were reported in Asia [2]. Keratitis is another example of a discriminatory infectious disease. Keratitis rates were considerably higher in countries such as Nepal, Pakistan, Thailand, Egypt, and Mexico [2]. These countries are an apparent representation that such disease rates could be related to healthcare system failures, resource depletion, unavailability of diagnostic tests, and impoverished policies in infection control, as discussed by Bongomin et al. (2017) [2].

HIV-associated fungal diseases represent one of the greatest challenges in Africa. Diseases such as cryptococcosis, candidiasis, histoplasmosis, and emergomycosis are among Africa’s most affected HIV-associated diseases despite the implementation of antiretroviral therapy [135]. Recent data published by UNAIDS showed that among the 37.7 million people living with HIV worldwide, more than 25 million live in Africa [136]. These data are alarming, considering immunosuppression is a risk factor for fungal infections. Notably, fungal diseases can no longer be neglected at the expense of other diseases [137].

It is also worth mentioning that a parallel could be drawn regarding mycosis incidents worldwide, as shown in Table 1. It can be observed that HIV/AIDS appears as a frequent and critical condition in cryptococcal infections, whereas long-term ICU and cancer/immunosuppression have a greater association with candidemia and aspergillosis occurrences [138,139,140,141,142,143]. This is a remarkable point already described by literature towards elucidating epidemiological traits on which mycoses are based, highlighting new perspectives on the research of pathogenesis and immunology regarding fungal infections.

The worldwide strategy to fight fungal infections relies on developing new antifungal drugs to eradicate diseases that cause many annual deaths. The scientific community must design methodologies to circumvent the challenges imposed by neglecting illnesses that do not democratically affect the world. This is pivotal in countries where access to clinical resources is lacking. Thus, preventing diseases caused by fungi worldwide would help avoid millions of deaths caused by obscurantism.

## Data Availability

Not applicable.

## References

[1] Fausto A., Rodrigues M.L., Coelho C. (2019). The Still Underestimated Problem of Fungal Diseases Worldwide. Front. Microbiol..

[2] Bongomin F., Gago S., Oladele R.O., Denning D.W. (2017). Global and Multi-National Prevalence of Fungal Diseases—Estimate Precision. J. Fungi.

[3] Berman J., Krysan D.J. (2020). Drug Resistance and Tolerance in Fungi. Nat. Rev. Microbiol..

[4] Linder K.A., Kauffman C.A. (2019). Histoplasmosis: Epidemiology, Diagnosis, and Clinical Manifestations. Curr. Fungal Infect. Rep..

[5] Crum N.F. (2022). Coccidioidomycosis: A Contemporary Review. Infect. Dis. Ther..

[6] Nucci M., Anaissie E. (2007). Fusarium Infections in Immunocompromised Patients. Clin. Microbiol. Rev..

[7] Cheng V.C.C., Chan J.F.W., Ngan A.H.Y., To K.K.W., Leung S.Y., Tsoi H.W., Yam W.C., Tai J.W.M., Wong S.S.Y., Tse H. (2009). Outbreak of Intestinal Infection Due to *Rhizopus microsporus*. J. Clin. Microbiol..

[8] Bowers J.R., Monroy-Nieto J., Gade L., Travis J., Refojo N., Abrantes R., Santander J., French C., Cecilia Dignani M., Ines Hevia A. (2020). *Rhizopus microsporus* Infections Associated with Surgical Procedures, Argentina, 2006–2014. Emerg. Infect. Dis..

[9] Samaddar A., Sharma A. (2021). Emergomycosis, an Emerging Systemic Mycosis in Immunocompromised Patients: Current Trends and Future Prospects. Front. Med..

[10] Ciurea C.N., Kosovski I.B., Mare A.D., Toma F., Pintea-Simon I.A., Man A. (2020). *Candida* and Candidiasis—Opportunism versus Pathogenicity: A Review of the Virulence Traits. Microorganisms.

[11] Lass-Flörl C. (2018). Treatment of Infections Due to *Aspergillus terreus* Species Complex. J. Fungi.

[12] Latgé J.P., Chamilos G. (2020). *Aspergillus fumigatus* and Aspergillosis in 2019. Clin. Microbiol. Rev..

[13] Rudramurthy S.M., Paul R.A., Chakrabarti A., Mouton J.W., Meis J.F. (2019). Invasive Aspergillosis by *Aspergillus flavus*: Epidemiology, Diagnosis, Antifungal Resistance, and Management. J. Fungi.

[14] Firacative C., Trilles L., Meyer W. (2022). Recent Advances in *Cryptococcus* and Cryptococcosis. Microorganisms.

[15] Martinez-Rossi N.M., Peres N.T.A., Bitencourt T.A., Martins M.P., Rossi A. (2021). State-of-the-Art Dermatophyte Infections: Epidemiology Aspects, Pathophysiology, and Resistance Mechanisms. J. Fungi.

[16] Azor M., Gené J., Cano J., Guarro J. (2007). Universal in vitro Antifungal Resistance of Genetic Clades of the *Fusarium solani* Species Complex. Antimicrob. Agents Chemother..

[17] Garcia-Rubio R., Cuenca-Estrella M., Mellado E. (2017). Triazole Resistance in *Aspergillus* Species: An Emerging Problem. Drugs.

[18] Yousfi H., Ranque S., Rolain J.M., Bittar F. (2019). In Vitro Polymyxin Activity against Clinical Multidrug-Resistant Fungi. Antimicrob. Resist. Infect. Control.

[19] Yoon Y.K., Yang K.S., Kim J., Moon C., Lee M.S., Hur J., Kim J.Y., Kim S.W. (2021). Clinical Implications of Multidrug-Resistant Microorganisms and Fungi Isolated from Patients with Intra-Abdominal Infections in the Republic of Korea: A Multicenter Study. Diagn. Microbiol. Infect. Dis..

[20] Sanglard D., Ischer F., Bille J. (2001). Role of ATP-Binding-Cassette Transporter Genes in High-Frequency Acquisition of Resistance to Azole Antifungals in *Candida glabrata*. Antimicrob. Agents Chemother..

[21] Tobin M.B., Peery R.B., Skatrud P.L. (1997). Genes Encoding Multiple Drug Resistance-Like Proteins in *Aspergillus fumigatus* and *Aspergillus flavus*. Gene.

[22] Prakash A., Sharma C., Singh A., Kumar Singh P., Kumar A., Hagen F., Govender N.P., Colombo A.L., Meis J.F., Chowdhary A. (2016). Evidence of Genotypic Diversity among *Candida auris* Isolates by Multilocus Sequence Typing, Matrix-Assisted Laser Desorption Ionization Time-of-Flight Mass Spectrometry and Amplified Fragment Length Polymorphism. Clin. Microbiol. Infect..

[23] Thompson G.R., Wiederhold N.P., Vallor A.C., Villareal N.C., Lewis J.S., Patterson T.F. (2008). Development of Caspofungin Resistance Following Prolonged Therapy for Invasive Candidiasis Secondary to *Candida glabrata* Infection. Antimicrob. Agents Chemother..

[24] Houšť J., Spížek J., Havlíček V. (2020). Antifungal Drugs. Metabolites.

[25] Campoy S., Adrio J.L. (2017). Antifungals. Biochem. Pharmacol..

[26] Dancer S.J. (2014). Controlling Hospital-Acquired Infection: Focus on the Role of the Environment and New Technologies for Decontamination. Clin. Microbiol. Rev..

[27] Sherertz R.J., Belani A., Kramer B.S., Elfenbein G.J., Weiner R.S., Sullivan M.L., Ronald Thomas R.G., Gregory Samsa F.P., Hill C., Carolina N. (1987). Impact of Air Filtration on Nosocomial *Aspergillus* Infections Unique Risk of Bone Marrow Transplant Recipients. Am. J. Med..

[28] Oren I., Haddad N., Finkelstein R., Rowe J.M. (2001). Invasive Pulmonary Aspergillosis in Neutropenic Patients during Hospital Construction: Before and after Chemoprophylaxis and Institution of HEPA Filters. Am. J. Hematol..

[29] Hu S., Zhu F., Jiang W., Wang Y., Quan Y., Zhang G., Gu F., Yang Y. (2021). Retrospective Analysis of the Clinical Characteristics of *Candida auris* Infection Worldwide from 2009 to 2020. Front. Microbiol..

[30] Chowdhary A., Sharma C., Meis J.F. (2017). *Candida auris*: A Rapidly Emerging Cause of Hospital-Acquired Multidrug-Resistant Fungal Infections Globally. PLoS Pathog..

[31] Satoh K., Makimura K., Hasumi Y., Nishiyama Y., Uchida K., Yamaguchi H. (2009). *Candida auris* sp. nov., a Novel Ascomycetous Yeast Isolated from the External Ear Canal of an Inpatient in a Japanese Hospital. Microbiol. Immunol..

[32] Schelenz S., Hagen F., Rhodes J.L., Abdolrasouli A., Chowdhary A., Hall A., Ryan L., Shackleton J., Trimlett R., Meis J.F. (2016). First Hospital Outbreak of the Globally Emerging *Candida auris* in a European Hospital. Antimicrob. Resist. Infect. Control.

[33] De Cássia Orlandi Sardi J., Silva D.R., Soares Mendes-Giannini M.J., Rosalen P.L. (2018). *Candida auris*: Epidemiology, Risk Factors, Virulence, Resistance, and Therapeutic Options. Microb. Pathog..

[34] Du H., Bing J., Hu T., Ennis C.L., Nobile C.J., Huang G. (2020). *Candida auris*: Epidemiology, Biology, Antifungal Resistance, and Virulence. PLoS Pathog..

[35] Rudramurthy S.M., Chakrabarti A., Paul R.A., Sood P., Kaur H., Capoor M.R., Kindo A.J., Marak R.S.K., Arora A., Sardana R. (2017). *Candida auris* Candidaemia in Indian ICUs: Analysis of Risk Factors. J. Antimicrob. Chemother..

[36] Zhu Y., O’Brien B., Leach L., Clarke A., Bates M., Adams E., Ostrowsky B., Quinn M., Dufort E., Southwick K. (2020). Laboratory Analysis of a *Candida auris* Outbreak Provides New Insights into an Emerging Pathogen. J. Clin. Microbiol..

[37] Lu P.L., Liu W.L., Lo H.J., der Wang F., Ko W.C., Hsueh P.R., Ho M.W., Liu C.E., Chen Y.H., Chen Y.C. (2018). Are We Ready for the Global Emergence of Multidrug-Resistant *Candida auris* in Taiwan?. J. Formos. Med. Assoc..

[38] Lockhart S.R., Etienne K.A., Vallabhaneni S., Farooqi J., Chowdhary A., Govender N.P., Colombo A.L., Calvo B., Cuomo C.A., Desjardins C.A. (2017). Simultaneous Emergence of Multidrug-Resistant *Candida auris* on 3 Continents Confirmed by Whole-Genome Sequencing and Epidemiological Analyses. Clin. Infect. Dis..

[39] Costa-Orlandi C.B., Sardi J.C.O., Pitangui N.S., de Oliveira H.C., Scorzoni L., Galeane M.C., Medina-Alarcón K.P., Melo W.C.M.A., Marcelino M.Y., Braz J.D. (2017). Fungal Biofilms and Polymicrobial Diseases. J. Fungi.

[40] Fanning S., Mitchell A.P. (2012). Fungal Biofilms. PLoS Pathog..

[41] Sherry L., Ramage G., Kean R., Borman A., Johnson E.M., Richardson M.D., Rautemaa-Richardson R. (2017). Biofilm-Forming Capability of Highly Virulent, Multidrug-Resistant *Candida auris*. Emerg. Infect. Dis..

[42] Larkin E., Hager C., Chandra J., Mukherjee P.K., Retuerto M., Salem I., Long L., Isham N., Kovanda L., Borroto-Esoda K. (2017). The Emerging Pathogen *Candida auris*: Growth Phenotype, Virulence Factors, Activity of Antifungals, and Effect of SCY-078, a Novel Glucan Synthesis Inhibitor, on Growth Morphology and Biofilm Formation. Antimicrob. Agents Chemother..

[43] Osei Sekyere J. (2018). *Candida auris*: A Systematic Review and Meta-Analysis of Current Updates on an Emerging Multidrug-Resistant Pathogen. Microbiologyopen.

[44] Aljindan R., Aleraky D.M., Mahmoud N., Abdalhamid B., Almustafa M., Abdulazeez S., Francis Borgio J. (2021). Drug Resistance-Associated Mutations in Erg11 of Multidrug-Resistant *Candida auris* in a Tertiary Care Hospital of Eastern Saudi Arabia. J. Fungi.

[45] Chowdhary A., Prakash A., Sharma C., Kordalewska M., Kumar A., Sarma S., Tarai B., Singh A., Upadhyaya G., Upadhyay S. (2018). A Multicentre Study of Antifungal Susceptibility Patterns among 350 *Candida auris* Isolates (2009-17) in India: Role of the ERG11 and FKS1 Genes in Azole and Echinocandin Resistance. J. Antimicrob. Chemother..

[46] Sharma D., Paul R.A., Rudramurthy S.M., Kashyap N., Bhattacharya S., Soman R., Shankarnarayan S.A., Chavan D., Singh S., Das P. (2022). Impact of FKS1 Genotype on Echinocandin In Vitro Susceptibility in *Candida auris* and In Vivo Response in a Murine Model of Infection. Antimicrob. Agents Chemother..

[47] Chekiri-Talbi M., Denning D.W. (2017). Burden of Fungal Infections in Algeria. Eur. J. Clin. Microbiol. Infect. Dis..

[48] Mandengue C.E., Denning D.W. (2018). The Burden of Serious Fungal Infections in Cameroon. J. Fungi.

[49] Zaki S.M., Denning D.W. (2017). Serious Fungal Infections in Egypt. Eur. J. Clin. Microbiol. Infect. Dis..

[50] Guto J.A., Bii C.C., Denning D.W. (2016). Estimated Burden of Fungal Infections in Kenya. J. Infect. Dev. Ctries..

[51] Kalua K., Zimba B., Denning D.W. (2018). Estimated Burden of Serious Fungal Infections in Malawi. J. Fungi.

[52] Sacarlal J., Denning D.W. (2018). Estimated Burden of Serious Fungal Infections in Mozambique. J. Fungi.

[53] Dunaiski C.M., Denning D.W. (2019). Estimated Burden of Fungal Infections in Namibia. J. Fungi.

[54] Riera F.O., Caeiro J.P., Denning D.W. (2018). Burden of Serious Fungal Infections in Argentina. J. Fungi.

[55] Giacomazzi J., Baethgen L., Carneiro L.C., Millington M.A., Denning D.W., Colombo A.L., Pasqualotto A.C. (2016). The Burden of Serious Human Fungal Infections in Brazil. Mycoses.

[56] Gugnani H.C., Denning D.W. (2016). Burden of Serious Fungal Infections in the Dominican Republic. J. Infect. Public Health.

[57] Bustamante B., Denning D.W., Campos P.E. (2017). Serious Fungal Infections in Peru. Eur. J. Clin. Microbiol. Infect. Dis..

[58] Gugnani H.C., Denning D.W., Rahim R., Sadat A., Belal M., Mahbub M.S. (2017). Burden of Serious Fungal Infections in Bangladesh. Eur. J. Clin. Microbiol. Infect. Dis..

[59] Zhou L.H., Jiang Y.K., Li R.Y., Huang L.P., Yip C.W., Denning D.W., Zhu L.P. (2020). Risk-Based Estimate of Human Fungal Disease Burden, China. Emerg. Infect. Dis..

[60] Ray A., Aayilliath K.A., Banerjee S., Chakrabarti A., Denning D.W. (2022). Burden of Serious Fungal Infections in India. Open Forum Infect. Dis..

[61] Wahyuningsih R., Adawiyah R., Sjam R., Prihartono J., Ayu Tri Wulandari E., Rozaliyani A., Ronny R., Imran D., Tugiran M., Siagian F.E. (2021). Serious Fungal Disease Incidence and Prevalence in Indonesia. Mycoses.

[62] Huh K., Ha Y.E., Denning D.W., Peck K.R. (2017). Serious Fungal Infections in Korea. Eur. J. Clin. Microbiol. Infect. Dis..

[63] Turdumambetova G.K., Osmanov A., Denning D.W. (2019). The Burden of Serious Fungal Infections in Kyrgyzstan. J. Fungi.

[64] Velayuthan R.D., Samudi C., Singh H.K.L., Ng K.P., Shankar E.M., Denning D.W. (2018). Estimation of the Burden of Serious Human Fungal Infections in Malaysia. J. Fungi.

[65] Jabeen K., Farooqi J., Mirza S., Denning D., Zafar A. (2017). Serious Fungal Infections in Pakistan. Eur. J. Clin. Microbiol. Infect. Dis..

[66] Bobokhojaev O.I., Osmanov A., Aliev S.P., Radjabzoda A.S., Avgonov Z.T., Manonov S.T., Denning D.W. (2019). The Burden of Serious Fungal Infections in Tajikistan. J. Fungi.

[67] Huseynov R.M., Javadov S.S., Osmanov A., Khasiyev S., Valiyeva S.R., Almammadova E., Denning D.W. (2021). The Burden of Serious Fungal Infections in Azerbaijan. Ther. Adv. Infect. Dis..

[68] Lagrou K., Maertens J., van Even E., Denning D.W. (2015). Burden of Serious Fungal Infections in Belgium. Mycoses.

[69] Gamaletsou M.N., Drogari-Apiranthitou M., Denning D.W., Sipsas N.V. (2016). An Estimate of the Burden of Serious Fungal Diseases in Greece. Eur. J. Clin. Microbiol. Infect. Dis..

[70] Arsenijević V.A., Denning D.W. (2018). Estimated Burden of Serious Fungal Diseases in Serbia. J. Fungi.

[71] Özenci V., Klingspor L., Ullberg M., Chryssanthou E., Denning D.W., Kondori N. (2019). Estimated Burden of Fungal Infections in Sweden. Mycoses.

[72] Gao J., Chow E.W.L., Wang H., Xu X., Cai C., Song Y., Wang J., Wang Y. (2021). LncRNA DINOR Is a Virulence Factor and Global Regulator of Stress Responses in *Candida auris*. Nat. Microbiol..

[73] Zaragoza O. (2019). Basic Principles of the Virulence of *Cryptococcus*. Virulence.

[74] Maziarz E.K., Perfect J.R. (2016). Cryptococcosis. Infect. Dis. Clin. N. Am..

[75] Garcia-Hermoso D., Janbon G., Dromer F. (1999). Epidemiological Evidence for Dormant *Cryptococcus neoformans* Infection. J. Clin. Microbiol..

[76] Lindell D.M., Ballinger M.N., McDonald R.A., Toews G.B., Huffnagle G.B. (2006). Immunologic Homeostasis during Infection: Coexistence of Strong Pulmonary Cell-Mediated Immunity to Secondary *Cryptococcus neoformans* Infection While the Primary Infection Still Persists at Low Levels in the Lungs. J. Immunol..

[77] Baker R., Haugen R. (1955). Tissue Changes and Tissue Diagnosis in Cryptococcosis. Am. J. Clin. Pathol..

[78] Schwartz D. (1988). Characterization of the Biological Activity of *Cryptococcus* Infections in Surgical Pathology. The Budding Index and Carminophilic Index. Ann. Clin. Lab. Sci..

[79] Iyer K.R., Revie N.M., Fu C., Robbins N., Cowen L.E. (2021). Treatment Strategies for Cryptococcal Infection: Challenges, Advances and Future Outlook. Nat. Rev. Microbiol..

[80] De Oliveira H.C., Rossi S.A., García-Barbazán I., Zaragoza Ó., Trevijano-Contador N. (2021). Cell Wall Integrity Pathway Involved in Morphogenesis, Virulence and Antifungal Susceptibility in *Cryptococcus neoformans*. J. Fungi.

[81] Garcia-Rubio R., de Oliveira H.C., Rivera J., Trevijano-Contador N. (2020). The Fungal Cell Wall: *Candida*, *Cryptococcus*, and *Aspergillus* Species. Front. Microbiol..

[82] Cao C., Wang Y., Husain S., Soteropoulos P., Xue C. (2019). A Mechanosensitive Channel Governs Lipid Flippase-Mediated Echinocandin Resistance in *Cryptococcus neoformans*. mBio.

[83] Chang M., Sionov E., Lamichhane A.K., Kwon-chung K.J., Chang Y.C. (2018). Crossm Roles of Three *Cryptococcus neoformans* and *Cryptococcus gattii* Efflux Pump-Coding Genes in Response to Drug Treatment. Antimicrob. Agents Chemother..

[84] Sionov E., Chang Y.C., Garraffo H.M., Dolan M.A., Ghannoum M.A., Kwon-Chung K.J. (2012). Identification of a *Cryptococcus neoformans* Cytochrome P450 Lanosterol 14α-Demethylase (Erg11) Residue Critical for Differential Susceptibility between Fluconazole/Voriconazole and Itraconazole/Posaconazole. Antimicrob. Agents Chemother..

[85] Yang M.L., Uhrig J., Vu K., Singapuri A., Dennis M., Gelli A., Thompson G.R. (2016). Fluconazole Susceptibility in *Cryptococcus gattii* Is Dependent on the ABC Transporter Pdr11. Antimicrob. Agents Chemother..

[86] Zafar H., Altamirano S., Ballou E.R., Nielsen K. (2019). A Titanic Drug Resistance Threat in *Cryptococcus neoformans*. Curr. Opin. Microbiol..

[87] Zaragoza O., Rocío G.R., Nosanchuk J.D., Cuenca-Estrella M., Rodríguez-Tudela J.L., Casadevall A. (2010). Fungal Cell Gigantism during Mammalian Infection. PLoS Pathog..

[88] Gerstein A.C., Fu M.S., Mukaremera L., Li Z., Ormerod K.L., Fraser J.A., Berman J., Nielsen K. (2015). Polyploid Titan Cells Produce Haploid and Aneuploid Progeny to Promote Stress Adaptation. mBio.

[89] Carlson T., Lupinacci E., Moseley K., Chandrasekaran S. (2021). Effects of Environmental Factors on Sensitivity of *Cryptococcus neoformans* to Fluconazole and Amphotericin B. FEMS Microbiol. Lett..

[90] Russo A., Tiseo G., Falcone M., Menichetti F. (2020). Pulmonary Aspergillosis: An Evolving Challenge for Diagnosis and Treatment. Infect. Dis. Ther..

[91] Cadena J., Thompson G.R., Patterson T.F. (2021). Aspergillosis: Epidemiology, Diagnosis, and Treatment. Infect. Dis. Clin. N. Am..

[92] Cornillet A., Camus C., Nimubona S., Gandemer V., Tattevin P., Belleguic C., Chevrier S., Meunier C., Lebert C., Aupée M. (2006). Comparison of Epidemiological, Clinical, and Biological Features of Invasive Aspergillosis in Neutropenic and Nonneutropenic Patients: A 6-Year Survey. Clin. Infect. Dis..

[93] Eigl S., Prattes J., Lackner M., Willinger B., Spiess B., Reinwald M., Selitsch B., Meilinger M., Neumeister P., Reischies F. (2015). Multicenter Evaluation of a Lateral-Flow Device Test for Diagnosing Invasive Pulmonary Aspergillosis in ICU Patients. Crit. Care.

[94] Lahmer T., Kriescher S., Herner A., Rothe K., Spinner C.D., Schneider J., Mayer U., Neuenhahn M., Hoffmann D., Geisler F. (2021). Invasive Pulmonary Aspergillosis in Critically Ill Patients with Severe COVID-19 Pneumonia: Results from the Prospective AspCOVID-19 Study. PLoS ONE.

[95] Koehler P., Cornely O.A., Böttiger B.W., Dusse F., Eichenauer D.A., Fuchs F., Hallek M., Jung N., Klein F., Persigehl T. (2020). COVID-19 Associated Pulmonary Aspergillosis. Mycoses.

[96] Taccone F.S., van den Abeele A.M., Bulpa P., Misset B., Meersseman W., Cardoso T., Paiva J.A., Blasco-Navalpotro M., de Laere E., Dimopoulos G. (2015). Epidemiology of Invasive Aspergillosis in Critically Ill Patients: Clinical Presentation, Underlying Conditions, and Outcomes. Crit. Care.

[97] Dunne K., Hagen F., Pomeroy N., Meis J.F., Rogers T.R. (2017). Intercountry Transfer of Triazole-Resistant *Aspergillus fumigatus* on Plant Bulbs. Clin. Infect. Dis..

[98] Sharpe A.R., Lagrou K., Meis J.F., Chowdhary A., Lockhart S.R., Verweij P.E. (2018). Triazole Resistance Surveillance in *Aspergillus fumigatus*. Med. Mycol..

[99] Meis J.F., Chowdhary A., Rhodes J.L., Fisher M.C., Verweij P.E. (2016). Clinical Implications of Globally Emerging Azole Resistance in *Aspergillus fumigatus*. Philos. Trans. R. Soc. B Biol. Sci..

[100] Brauer V.S., Rezende C.P., Pessoni A.M., de Paula R.G., Rangappa K.S., Nayaka S.C., Gupta V.K., Almeida F. (2019). Antifungal Agents in Agriculture: Friends and Foes of Public Health. Biomolecules.

[101] Hagiwara D., Arai T., Takahashi H., Kusuya Y., Watanabe A., Kamei K. (2018). Non-Cyp51A Azole-Resistant *Aspergillus fumigatus* Isolates with Mutation in HMG-CoA Reductase. Emerg. Infect. Dis..

[102] Handelman M., Morogovsky A., Liu W., Ben-Ami R., Osherov N. (2021). Triazole-Resistant *Aspergillus fumigatus* in an Israeli Patient with Chronic Cavitary Pulmonary Aspergillosis Due to a Novel E306K Substitution in Hmg1. Antimicrob. Agents Chemother..

[103] Pinto e Silva A., Miranda I.M., Branco J., Oliveira P., Faria-Ramos I., Silva R.M., Rodrigues A.G., Costa-de-Oliveira S. (2020). FKS1 Mutation Associated with Decreased Echinocandin Susceptibility of *Aspergillus fumigatus* Following Anidulafungin Exposure. Sci. Rep..

[104] Satish S., Perlin D.S. (2019). Echinocandin Resistance in *Aspergillus fumigatus* Has Broad Implications for Membrane Lipid Perturbations That Influence Drug-Target Interactions. Microbiol. Insights.

[105] Prestel C., Anderson E., Forsberg K., Lyman M., de Perio M.A., Kuhar D., Edwards K., Rivera M., Shugart A., Walters M. (2021). *Candida auris* Outbreak in a COVID-19 Specialty Care Unit—Florida, July–August 2020. Morb. Mortal. Wkly. Rep..

[106] Chowdhary A., Tarai B., Singh A., Sharma A. (2020). Multidrug-Resistant *Candida auris* Infections in Critically Ill Coronavirus Disease Patients, India, April–July 2020. Emerg. Infect. Dis..

[107] Allaw F., Zahreddine N.K., Ibrahim A., Tannous J., Taleb H., Bizri A.R., Dbaibo G., Kanj S.S. (2021). First *Candida auris* Outbreak during a Covid-19 Pandemic in a Tertiary-Care Center in Lebanon. Pathogens.

[108] Bölükbaşi Y., Erköse Genç G., Orhun G., Kuşkucu M.A., Çağatay A., Önel M., Öngen B., AğaçfiDan A., Esen F., Erturan Z. (2021). First Case of COVID-19 Positive *Candida auris* Fungemia in Turkey. Mikrobiyol. Bul..

[109] De Almeida J.N., Francisco E.C., Hagen F., Brandão I.B., Pereira F.M., Presta Dias P.H., de Miranda Costa M.M., de Souza Jordão R.T., de Groot T., Colombo A.L. (2021). Emergence of *Candida auris* in Brazil in a COVID-19 Intensive Care Unit. J. Fungi.

[110] Martins A.C., Psaltikidis E.M., de Lima T.C., Fagnani R., Schreiber A.Z., de Oliveira Conterno L., Kamei K., Watanabe A., Trabasso P., Resende M.R. (2021). COVID-19 and Invasive Fungal Coinfections: A Case Series at a Brazilian Referral Hospital. J. Med. Mycol..

[111] Bartoletti M., Pascale R., Cricca M., Rinaldi M., Maccaro A., Bussini L., Fornaro G., Tonetti T., Pizzilli G., Francalanci E. (2021). Epidemiology of Invasive Pulmonary Aspergillosis among Intubated Patients with COVID-19: A Prospective Study. Clin. Infect. Dis..

[112] Marr K.A., Platt A., Tornheim J.A., Zhang S.X., Datta K., Cardozo C., Garcia-Vidal C. (2021). Aspergillosis Complicating Severe Coronavirus Disease. Emerg. Infect. Dis..

[113] Mohamed A., Hassan T., Trzos-Grzybowska M., Thomas J., Quinn A., O’Sullivan M., Griffin A., Rogers T.R., Talento A.F. (2021). Multi-Triazole-Resistant *Aspergillus fumigatus* and SARS-CoV-2 Co-Infection: A Lethal Combination. Med. Mycol. Case Rep..

[114] Azhar A., Khan W.H., Khan P.A., Alhosaini K., Owais M., Ahmad A. (2022). Mucormycosis and COVID-19 Pandemic: Clinical and Diagnostic Approach. J. Infect. Public Health.

[115] Hoenigl M., Seidel D., Carvalho A., Rudramurthy S.M., Arastehfar A., Gangneux J.P., Nasir N., Bonifaz A., Araiza J., Klimko N. (2022). The Emergence of COVID-19 Associated Mucormycosis: A Review of Cases from 18 Countries. Lancet Microbe.

[116] Chandley P., Subba P., Rohatgi S. (2022). COVID-19-Associated Mucormycosis: A Matter of Concern Amid the SARS-CoV-2 Pandemic. Vaccines.

[117] Pathakumari B., Liang G., Liu W. (2020). Immune Defence to Invasive Fungal Infections: A Comprehensive Review. Biomed. Pharmacother..

[118] Fernández-García O.A., Cuellar-Rodríguez J.M. (2021). Immunology of Fungal Infections. Infect. Dis. Clin. N. Am..

[119] Biswas P.S. (2021). Vaccine-Induced Immunological Memory in Invasive Fungal Infections—A Dream so Close yet so Far. Front. Immunol..

[120] Lee Y., Puumala E., Robbins N., Cowen L.E. (2021). Antifungal Drug Resistance: Molecular Mechanisms in Candida Albicans and beyond. Chem. Rev..

[121] Armstrong-James D., Brown G.D., Netea M.G., Zelante T., Gresnigt M.S., van de Veerdonk F.L., Levitz S.M. (2017). Immunotherapeutic Approaches to Treatment of Fungal Diseases. Lancet Infect. Dis..

[122] Pietrella D., Rachini A., Torosantucci A., Chiani P., Brown A.J.P., Bistoni F., Costantino P., Mosci P., d’Enfert C., Rappuoli R. (2010). A β-Glucan-Conjugate Vaccine and Anti-β-Glucan Antibodies Are Effective against Murine Vaginal Candidiasis as Assessed by a Novel in vivo Imaging Technique. Vaccine.

[123] Specht C.A., Lee C.K., Huang H., Tipper D.J., Shen Z.T., Lodge J.K., Leszyk J., Ostroff G.R., Levitz S.M. (2015). Protection against Experimental Cryptococcosis Following Vaccination with Glucan Particles Containing *Cryptococcus alkaline* Extracts. mBio.

[124] Clemons K.V., Danielson M.E., Michel K.S., Liu M., Ottoson N.C., Leonardo S.M., Martinez M., Chen V., Antonysamy M.A., Stevens D.A. (2014). Whole Glucan Particles as a Vaccine against Murine Aspergillosis. J. Med. Microbiol..

[125] Akhtar N., Joshi A., Kaushik V., Kumar M., Amin-ul Mannan M. (2021). In-Silico Design of a Multivalent Epitope-Based Vaccine against *Candida auris*. Microb. Pathog..

[126] Devi S.J.N., Schneerson R., Egan W., Ulrich T.J., Bryla D., Robbins J.B., Bennett J.E. (1991). *Cryptococcus neoformans* Serotype A Glucuronoxylomannan-Protein Conjugate Vaccines: Synthesis, Characterization, and Immunogenicity. Infect. Immun..

[127] Specht C.A., Homan E.J., Lee C.K., Mou Z., Gomez C.L., Hester M.M., Abraham A., Rus F., Ostroff G.R., Levitz S.M. (2022). Protection of Mice against Experimental Cryptococcosis by Synthesized Peptides Delivered in Glucan Particles. mBio.

[128] Hester M.M., Lee C.K., Abraham A., Khoshkenar P., Ostroff G.R., Levitz S.M., Specht C.A. (2020). Protection of Mice against Experimental Cryptococcosis Using Glucan Particle-Based Vaccines Containing Novel Recombinant Antigens. Vaccine.

[129] Gu X., Hua Y.H., Zhang Y.D., Bao D., Lv J., Hu H.F. (2021). The Pathogenesis of *Aspergillus fumigatus*, Host Defense Mechanisms, and the Development of AFMP4 Antigen as a Vaccine. Pol. J. Microbiol..

[130] Rayens E., Rabacal W., Kang S.E., Celia B.N., Momany M., Norris K.A. (2021). Vaccine-Induced Protection in Two Murine Models of Invasive Pulmonary Aspergillosis. Front. Immunol..

[131] Muthu V., Sehgal I.S., Dhooria S., Aggarwal A.N., Agarwal R. (2018). Utility of Recombinant *Aspergillus fumigatus* Antigens in the Diagnosis of Allergic Bronchopulmonary Aspergillosis: A Systematic Review and Diagnostic Test Accuracy Meta-Analysis. Clin. Exp. Allergy.

[132] Érez-Cantero A.P., Serrano D.R., Navarro-Rodríguez P., Schätzlein A.G., Uchegbu I.F., Torrado J.J., Capilla J. (2019). Increased Efficacy of Oral Fixed-Dose Combination of Amphotericin B and AHCC^®^ Natural Adjuvant against Aspergillosis. Pharmaceutics.

[133] Stephen-Victor E., Karnam A., Fontaine T., Beauvais A., Das M., Hegde P., Prakhar P., Holla S., Balaji K.N., Kaveri S.V. (2017). *Aspergillus fumigatus* Cell Wall A-(1,3)-Glucan Stimulates Regulatory T-Cell Polarization by Inducing PD-L1 Expression on Human Dendritic Cells. J. Infect. Dis..

[134] Wurster S., Robinson P., Albert N.D., Tarrand J.J., Goff M., Swamydas M., Lim J.K., Lionakis M.S., Kontoyiannis D.P. (2020). Protective Activity of Programmed Cell Death Protein 1 Blockade and Synergy with Caspofungin in a Murine Invasive Pulmonary Aspergillosis Model. J. Infect. Dis..

[135] Bongomin F., Fayemiwo S.A. (2021). Epidemiology of Fungal Diseases in Africa: A Review of Diagnostic Drivers. Curr. Med. Mycol..

[136] Unaids (2021). Unaids Data 2021.

[137] Rodrigues M.L., Nosanchuk J.D. (2020). Fungal Diseases as Neglected Pathogens: A Wake-up Call to Public Health Officials. PLoS Negl. Trop. Dis..

[138] Mirza S.A., Phelan M., Rimland D., Graviss E., Hamill R., Brandt M.E., Gardner T., Sattah M., de Leon G.P., Baughman W. (2003). The Changing Epidemiology of Cryptococcosis: An Update from Population-Based Active Surveillance in 2 Large Metropolitan Areas, 1992–2000. Clin. Infect. Dis..

[139] Sridhar H., Jayshree R.S., Bapsy P.P., Appaji L., Navin Kumar M., Shafiulla M., VijayKumar B.R. (2002). Invasive Aspergillosis in Cancer. Mycoses.

[140] Hankovszky P., Társy D., Öveges N., Molnár Z. (2015). Invasive Candida Infections in the ICU: Diagnosis and Therapy. J. Crit. Care Med..

[141] Poissy J., Rouzé A., Cornu M., Nseir S., Sendid B. (2022). The Changing Landscape of Invasive Fungal Infections in ICUs: A Need for Risk Stratification to Better Target Antifungal Drugs and the Threat of Resistance. J. Fungi.

[142] Limper A.H., Adenis A., Le T., Harrison T.S. (2017). Fungal Infections in HIV/AIDS. Lancet Infect. Dis..

[143] Sipsas N.V., Kontoyiannis D.P. (2012). Invasive Fungal Infections in Patients with Cancer in the Intensive Care Unit. Int. J. Antimicrob. Agents.

